# Attenuated inflammatory response of monocyte-derived macrophage from patients with BD: a preliminary report

**DOI:** 10.1186/s40345-019-0148-x

**Published:** 2019-06-01

**Authors:** Bruna M. Ascoli, Mariana M. Parisi, Giovana Bristot, Bárbara Antqueviezc, Luiza P. Géa, Rafael Colombo, Flávio Kapczinski, Fátima Theresinha Costa Rodrigues Guma, Elisa Brietzke, Florencia M. Barbé-Tuana, Adriane R. Rosa

**Affiliations:** 1 Laboratory of Molecular Psychiatry, Hospital de Clínicas de Porto Alegre (HCPA), Rua Ramiro Barcelos, 2350, Porto Alegre, RS Brazil; 2 Postgraduate Program in Psychiatry and Behavioral Sciences, Universidade Federal do Rio Grande do Sul (UFRGS), Rua Ramiro Barcelos, 2400, Porto Alegre, RS Brazil; 3 Laboratory of Molecular Biology and Bioinformatics, Department of Biochemistry, UFRGS, Rua Ramiro Barcelos, 2600, Porto Alegre, Brazil; 4 Postgraduate Program in Biological Sciences: Biochemistry, UFRGS, Rua Ramiro Barcelos, 2600, Porto Alegre, RS Brazil; 5 Postgraduate Program in Biological Sciences: Pharmacology and Therapeutics, UFRGS, Rua Sarmento Leite 500, Porto Alegre, RS Brazil; 6 Laboratory of Pharmacology and Physiology, Universidade de Caxias do Sul (UCS), Rua Francisco Getúlio Vargas, 1130, Caxias Do Sul, RS Brazil; 7 Department of Psychiatry and Behavioural Neurosciences, McMaster University, 1280 Main Street West, Hamilton, ON Canada; 8 St. Joseph’s Healthcare Hamilton, 100 West 5th Street, Hamilton, ON Canada; 9 Laboratory of Biochemistry and Cellular Biology of Lipids, Department of Biochemistry, UFRGS, Rua Ramiro Barcelos, 2600, Porto Alegre, RS Brazil; 10 Mood Disorders Molecular and Behavioral Neurosciences Research Group, Department of Psychiatry, Universidade Federal de São Paulo (USP), Rua Sena Madureira, 1500, São Paulo, SP Brazil; 11 Postgraduate Program in Cellular and Molecular Biology, School of Sciences, Pontifícia Universidade Católica do Rio Grande do Sul (PUCRS), Avenida Ipiranga, 6681, Porto Alegre, RS Brazil

**Keywords:** Bipolar disorder, Mood disorders, Inflammatory cytokines, Macrophage polarization, Macrophage dysfunction

## Abstract

**Background:**

Innate immune system dysfunction has been recognized as an important element in the pathophysiology of bipolar disorder (BD). We aimed to investigate whether there are differences in the response of macrophages derived from patients in the early stages and late stages of BD and healthy subjects.

**Methods:**

Human monocytes purified from peripheral blood mononuclear cells (PBMCs) of patients with BD type I (n = 18)—further classified into early- and late stage BD patients according to their functioning- and from healthy individuals (n = 10) were differentiated into macrophages in vitro. Monocyte-derived macrophages (M) were exposed to IFNγ plus LPS-M(IFNγ + LPS)- or IL-4-M(IL-4)—to induce their polarization into the classical (also called M1) or alternative (also called M2) activation phenotypes, respectively; or either M*ψ* were not exposed to any stimuli characterizing the resting state (denominated M0). In vitro secretion of cytokines, such as IL-1β, IL-6, IL-10, and TNF-α, was used as an index of macrophage activity.

**Results:**

M(IFNγ + LPS) from late-stage BD patients produced less amount of IL-1β, IL-6, and IL-10 when compared to early-stage BD patients and healthy controls. Following alternative activation, M(IL-4) derived from late-stage patients secreted less IL-6 compared to the other groups. TNFα was less secreted by all macrophage phenotypes derived from late-stage patients when compared to healthy controls only (p < 0.005). M*ψ* from late-stage patients exhibited lower production of IL-1β and IL-10 compared to macrophages from healthy subjects and early-stage patients respectively. Interestingly, cytokines secretion from M(IFNγ + LPS), M(IL-4) and M*ψ* were similar between early-stage patients and healthy controls.

**Conclusion:**

Our results suggest a progressive dysfunction in the response of peripheral innate immune cells of BD patients in the late stages of the illness. This failure in the regulation of the immune system function may be implicated in the multisystemic progression of BD.

## Introduction

A remarkable recent shift in the understanding of bipolar disorder (BD) pathophysiology was the systematic documentation of its clinical and neurobiological progression (Berk et al. 2017; Kapczinski et al. 2014). At least for a large subgroup of patients, BD natural history, especially when not adequately treated, follows a progressive course with more episodes being associated with cognitive deficits, structural brain changes and treatment resistance (Bauer et al. 2017; Kessing and Andersen 2017). However, the validity of different methods that enable to discriminate in which point of the clinical and neurobiological progression of BD each patient would it remain under debate (Kapczinski et al. 2009). Nevertheless, taking into account ecological validity, instruments focused on functionality naturally emerge as a potentially useful tool. For instance, the Functioning Assessment Short Test (FAST) as previously described by Rosa et al. (2014), was used to discriminate between patients at early and late stages of BD. Early-stage BD patients were defined as exhibiting a mean FAST score lower than 11, while late-stage patients were those with a mean FAST score higher than 40 (Bonnín et al. 2018).

In addition to clinical course worsening and brain-related variables progressive deterioration, BD also has been conceptualized as a multisystemic progressive disorder (Brietzke et al. 2018; Leclerc et al. 2018). Longer duration of illness and a higher number of manic and depressive episodes have been shown to continuously increase the risk for general medical comorbidities, especially those linked to metabolic and immune dysfunction. There is strong replicated evidence showing a positive association between the number of mood episodes, mainly of manic polarity, and insulin and glucose dysfunction (Mansur et al. 2016a). Besides, these peripheral abnormalities also have been linked to suboptimal functioning as well as functional deterioration in individuals with mood disorders (Lin et al. 2014; Mansur et al. 2016b; McIntyre et al. 2011).

While the biological underpinnings of multisystemic progression in BD remain not completely known, there is a growing body of evidence suggesting the involvement of an immune response dysfunction in the pathogenesis of BD (Beumer et al. 2012; Drexhage et al. 2011; Haarman et al. 2014; Snijders et al. 2016). Two recent meta-analyses have shown that individuals with BD have an average peripheral level of proinflammatory cytokines higher than healthy controls (Modabbernia et al. 2013; Munkholm et al. 2013), but findings are heterogeneous (Boufidou et al. 2004; Brietzke et al. 2009; Guloksuz et al. 2010). Possible reasons for this heterogeneity may be attributed to non-controlled selection biases, as well as to the biological complexity of BD itself (Drexhage et al. 2010). Notwithstanding, the simple measurement of peripheral cytokines levels offers limited information about the functionality of the immune system in BD. Issues such as a large proportion of cases with subthreshold levels of cytokines, and especially the lack of information about which cell types could be the source of the cytokines may be better elucidated with in vitro studies (Rizzo et al. 2018). However, these studies are both relatively rare, and most of them do not integrate variables related to the clinical course of the disorder (Alcocer-Gómez et al. 2017). Hence, studies focusing on the main cellular producers of these cytokines—such as endothelial cells, monocytes, monocyte-derived dendritic cells, macrophages, and T cells—may represent an alternative approach to find stable markers for the immune response in BD (Becking et al. 2015).

Macrophages, as well as their counterparts in the central nervous system (CNS), the microglia, are essential components of the innate immune system, involved in the initiation and progression of various inflammatory and autoimmune diseases (Hammer et al. 2017). These cells have remarkable plasticity, as they switch and interconvert into different phenotypes and acquire functional characteristics, according to local cytokine milieu. In the presence of an inflammatory stimulus, such as lipopolysaccharide (LPS), interferon (IFN) and tumor necrosis factor (TNF), epigenetic changes in macrophages lead them to acquire a specific phenotype, associated with augmented secretion of proinflammatory cytokines and functional enhanced killing activity, denominated classical activation or M1 phenotype (Franco and Fernández-Suárez 2015). Macrophages can also display opposite abilities and acquire tissue repair actions to promote tissue homeostasis through a resolving, repair or healing anti-inflammatory phenotype (Nakagawa and Chiba 2015), called alternative activation phenotype or M2 phenotype. However, additional polarization profiles are observed with mixed phenotypes and functions (Xue et al. 2014).

Macrophages have multiple functions. For instance, these cells are constantly sampling the environment through their damage- and pathogen-associated molecular patterns (DAMPs and PAMPs) receptors, and can switch from the heal to kill function (Mills et al. 2014), inducing a different transcriptome program (Beyer et al. 2012) for phagocytosis of pathogens and clearance of cell debris. Eventually, macrophages act as effectors cells of cell-mediated immunity by presenting antigens to T cells and producing different types of cytokines and chemokines. These cells are not only key players in the initiation of inflammation, but also orchestrate its resolution (Linton and Thoman 2014). However, these homeostatic and reparative functions can be changed by continuous insult, resulting in the association of macrophages with distinct pathologies (Shapouri-Moghaddam et al. 2018).

The “macrophage-T cell theory” in psychiatry was initially described in 1992 (Barbosa et al. 2014). According to this theory, chronically activated macrophages and T cells produce cytokines and inflammatory mediators, which destabilize the brain in such a way that genetic and environmental influences can precipitate psychiatric symptoms (Beumer et al. 2012; Drexhage et al. 2010). In this regard, Haarman et al. (2014) investigated an extensive set of monocyte gene expression in BD patients showing a possible relationship between pro-inflammatory gene expression profile and the course of the illness, especially in individuals with earlier age at onset and longer duration of BD illness. Other two gene-expression profiling studies detected higher expression of a coherent set of 34 gene transcripts in the circulating monocytes of BD patients, suggesting an inflammatory gene expression “biosignature” (Drexhage et al. 2010; Padmos et al. 2008). In particular, the overexpression of monocyte activation genes was evident in 60–70% of cases, most in patients with mania, current depression or active psychosis. More recently, we investigated whether biochemical changes in the serum from BD patients during episodes could modulate the phenotype of the U-937 monocytic cell line (Ferrari et al. 2018). Our observations showed a robust upregulation in the expression of interleukin (IL)-1β and TNF in the cells that were treated with serum from manic and depressive patients compared with those treated with serum from euthymic patients (Ferrari et al. 2018).

Based on the role of the immune system in the multisystemic progression of BD, this study aimed to preliminarily investigate, in a primary culture of monocyte-derived macrophages (M*ψ*) from BD patients and healthy controls, the macrophage response following polarization. Also, we aimed to further examine potential differences in this pattern of macrophages derived from early and late stages of BD, without the limitations of measuring peripheral levels of cytokines.

## Methods

### Participants

Outpatients were recruited from the Bipolar Disorders Program at the Hospital de Clínicas de Porto Alegre (HCPA, Brazil). Inclusion criteria were (a) age > 18 years, (b) fulfillment of DSM-IV criteria for bipolar I (SCID-I), and (c) meeting criteria of remission defined as a score < 7 on both the Hamilton Depression Rating Scale (17-HAM-D) (Hamilton 1960) and the Young Mania Rating Scale (YMRS) (Young et al. 1978) for at least 1 month previous to the assessment. All patients received pharmacological treatment. Exclusion criteria included a history of any acute or chronic infectious or inflammatory diseases, autoimmune diseases, any severe or uncompensated general medical comorbidity, use of anti-inflammatory or immunomodulatory medications, pregnancy and postpartum period.

The BD group (n = 18) was evaluated regarding the clinical stage using the FAST. This is a 24-item scale that allows us the assessment of six specific areas of functioning: autonomy, occupational functioning, cognitive functioning, financial issues, interpersonal relationships and leisure time. The overall FAST score ranges from 0 to 72, where higher scores indicate greater disability (Rosa et al. 2007, 2009). For this study, patients were classified in early and late stages according to FAST as previously described in Rosa et al. (2014). Therefore, early-stage BD (BD-E, n = 9) was defined when patients present with a FAST score ≤ 11, and late-stage BD (BD-L, n = 9) as those with a FAST score ≥ 40. The Cognitive Bipolar Rating Assessment (COBRA) was also used to assess cognitive complaints in both groups (Lima et al. 2018).

The control group (HC, n = 10) consisted of healthy volunteers who had no current or previous history as well as no first-degree family history of major psychiatric disorders, assessed by the non-patient version of the Structured Clinical Interview for DSM-IV (SCID). Also, healthy subjects did not use any psychotropic or other medication and were free of any relevant medical illness that might have affected inflammation status at least 2 weeks before blood withdrawal.

All participants provided written informed consent before their inclusion. Procedures were approved by the Institutional Review Board of *Hospital de Clínicas de Porto Alegre* (HCPA, project number 150396).

### Monocyte isolation from PBMCs

**Fig. 1:**
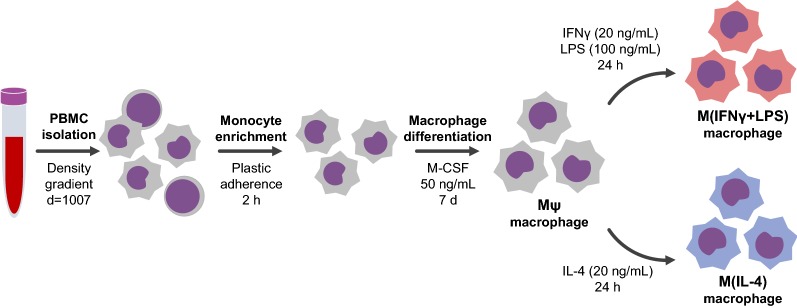
Workflow of monocyte isolation, and macrophage differentiation and polarization. Approximately 3–5 × 10^6^ PBMCs from early- and late-stage BD patients and healthy controls were seeded into 12-well cell culture plates and allowed to adhere for 2 h in a 5% CO_2_ incubator at 37 °C. Adherent cells were differentiated into macrophages in RPMI 1640 media supplemented with 10% FBS and M-CSF, 50 ng/mL for 7 days. For classical and alternative phenotypes, M*ψ* were incubated for additional 24 h with RPMI + 10% FBS supplemented with IFNγ (20 ng/mL) and LPS (100 ng/mL) or IL-4 (20 ng/mL), respectively

The procedures regarding monocyte isolation and macrophage differentiation were performed according to (Becker et al. 2015) and are summarized in Fig. 1. Approximately 40 mL of peripheral blood was obtained from patients and controls, and it was purified by density gradient with histopaque^®^-1077 (d = 1.077, Sigma Aldrich^®^) in the ratio of 1:1 and then centrifuged (400×*g* for 30 min in BRAKE OFF module). Peripheral blood mononuclear cells (PBMCs) were collected from the interphase, washed with phosphate-buffered saline (PBS, Sigma-Aldrich^®^) and resuspended in RPMI-1640 media (Invitrogen™) supplemented with 10% fetal bovine serum (FBS, Gibco), 200 mg/mL glutamine (Invitrogen™) and 100 U/mL penicillin 100 mg/mL streptomycin (Invitrogen™). Monocytes were purified from PBMCs by cell culture plastic adherence as follows: 3–5 × 10^6^ PBMCs per well were seeded into 12-well cell culture plates and allowed to adhere in a 5% CO_2_ incubator at 37 °C. After a 2-h incubation, non-adherent cells were removed. Adherent cells, mainly monocytes, were carefully washed twice with PBS and cultured for additional 7 days supplemented with macrophage colony-stimulating factor (M-CSF, 50 ng/mL, Peprotech) (Becker et al. 2015).

### Macrophage polarization

To induce polarization, M*ψ* were polarized towards the classical—mainly proinflammatory- or alternative—mainly anti-inflammatory—profiles (Ambarus et al. 2012; Becker et al. 2015; Solinas et al. 2010). In this regard, M*ψ* cultures were incubated for additional 24 h with RPMI + 10% FBS either supplemented with IFNγ (20 ng/mL, Peprotech) and LPS (100 ng/mL, Sigma-Aldrich^®^)—M(IFNγ + LPS)- or IL-4 (20 ng/mL, Peprotech)—M(IL-4)-, for the classical (M1) and alternative (M2) phenotypes, respectively.

### Cytokines secretion

After incubation, supernatants were collected and cytokines, such as IL-1β, IL-6, IL-10, and TNF-, were measured by multiplex assay using a Milliplex Map Human High Sensitivity T Cell Magnetic Bead Panel (HSTCMAG-28SK), according to the manufacturer’s instructions (Millipore, USA). Briefly, each diluted standard or quality control was added into the appropriate wells, and the assay buffer and macrophage culture supernatants were added to the sample wells. The magnetic beads were pipetted and the plates were sealed and incubated under agitation on the plate shaker for 16 h at 4 °C. Then, plates were washed three times and followed by the addition of detection antibodies into each well. After 1 h of incubation under agitation at room temperature, streptavidin conjugated to the fluorescent protein phycoerythrin was added and the plates were incubated for an additional 30 min at room temperature. Thereafter, plates were washed to remove the unbound streptavidin–phycoerythrin, sheath fluid was added to all wells and the beads were resuspended on the plate shaker for 5 min. The beads (minimum of 50 beads per cytokine) were analyzed in the Luminex^®^ 200TM instrument, which monitored the spectral properties of the beads while simultaneously measuring the amount of fluorescence associated with phycoerythrin. Raw data (median fluorescence intensity, MFI) was analyzed using a 5-parameter logistic method to determine concentrations of IL-1β, IL-6, IL-10 and TNFα (Luminex Xponent software 3.1).

Protein content from each culture was corrected using a standard curve from diluted bovine albumin serum (BSA) ranging from 0 to 70 µg/mL (Peterson 1979).

### Statistical analysis

Data were normalized using log-transformation, which was confirmed using Shapiro–Wilk’s test, and the homogeneity of variances was also confirmed by Levene’s test. Then, log-transformed data of each cytokine level from each polarization phenotype was analyzed using one-way ANOVA and considering staging (control, BD-E, and BD-L) as the independent variable. Post hoc comparisons were performed using Tukey and p < 0.05 were considered significant.

## Results

### Demographic and clinical features

Sample characteristics are shown in Table 1. No demographic differences were found among the three groups. As expected from the selection criteria, subjects with BD-L had a higher mean FAST score when compared to early-stage patients and healthy volunteers F = 50.874; p < 0.001).

**Table 1:** Characteristics of the sample

	Controls (n = 10)	BD-E (n = 9)	BD-L (n = 9)	F, t, x^2^	p value
Sex (female)^a^	7 (70)	8 (88.9)	6 (66.7)	1.39	0.498
Age (years)^b^	48 (14.34)	56.89 (12.23)	50.22 (14.25)	1.07	0.360
Years of education^b^	14.0 (4.69)	10.00 (3.08)	13.56 (5.96)	1.99	0.158
Age of bipolar diagnosis (years)^b^		43.11 (13.27)	35.56 (16.24)	1.08	0.296
Suicide attempts^a^		2 (22.2)	6 (66.7)	3.60	0.058
Number of hospitalizations^b^		1.44 (1.13)	3.33 (1.80)	− 2.66	*0.017*
BD type I^a^		7 (77.8)	8 (88.9)	0.40	0.527
HAM-D^b^		3.00 (1.73)	4.67 (2.12)	− 1.83	0.087
YMRS^b^		1.11 (1.96)	2.44 (2.83)	− 1.16	0.263
FAST^b^	5.10 (6.24)	6.33 (4.36)	45.55 (5.50)*	162.99	<* 0.001*
COBRA^b^	6.30 (5.98)	11.78 (10.08)	24.44 (10.14)*	10.34	*0.001*
Marital status^a^					
Married	5 (50)	3 (33.3)	3 (33.3)	4.70	0.583
Work situation^a^					
Employed	4 (40)	3 (33.3)	2 (22.2)	20.67	0.055
Medication^a^					
Lithium		3 (33.3)	4 (44.4)	0.23	0.629
Anticonvulsants		6 (66.7)	6 (66.7)	0	1
Antipsychotics		7 (77.8)	8 (88.9)	0.40	0.527
Antidepressants		3 (33.3)	4 (44.4)	0.23	0.629
Benzodiazepines		3 (33.3)	1 (11.1)	1.29	0.257

Student’s *t* test, ANOVA (followed by Tukey) or Chi square (v2) test

Italic values indicate of p value (< 0.05)

HAM-D, Hamilton Depression Scale; YMRS, Young Mania Rating Scale; FAST, Functioning Assessment Short Test; COBRA, Cognitive Complaints in Bipolar Disorder Rating Scale

* compared to BD-E and HC

^a^Data expressed as  %

^b^Data expressed as mean ± SD

### Lower secretion of cytokines by Mψ, M(IFNγ + LPS) and M(IL-4) derived from BD-L

For all pro-inflammatory cytokines levels, one-way ANOVA indicated differences among groups for M*ψ* (IL-1β, F_2,25_ = 3.99, p = 0.031; TNFα, F_2,25_ = 4.15, p = 0.028; and IL-6, F_2,25_ = 3.55, p = 0.044) and M(IFNγ + LPS) (IL-1β, F_2,25_ = 8.24, p = 0.002; TNFα, F_2,25_ = 7.98, p = 0.002; and IL-6, F_2,25_ = 7.44, p = 0.003) phenotypes. No differences were observed in the secretion of these cytokines by M(IL-4) phenotype (IL-1β, F_2,25_ = 0.74, p = 0.488; TNFα, F_2,25_ = 2.98, p = 0.069; and IL-6, F_2,25_ = 0.92, p = 0.413).

**Fig. 2:**
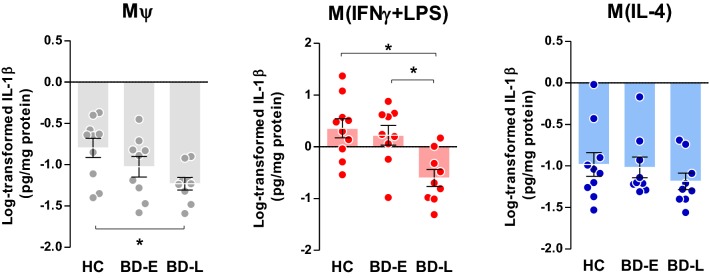
Secretion of IL-1β levels by different phenotypes of macrophages derived from individuals with BD at early (E) and late (L) stages and healthy controls (HC). One-way ANOVA followed by Tukey post hoc, data expressed by mean ± SEM, *p < 0.05

**Fig. 3:**
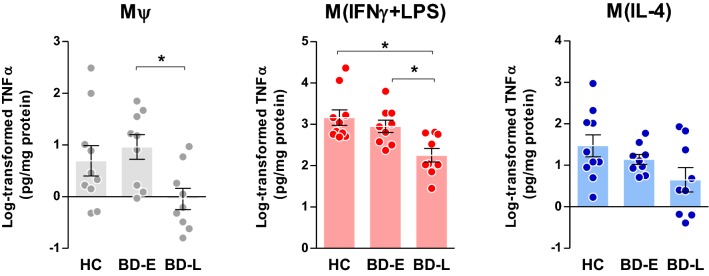
TNFα levels produced by different phenotypes of macrophages derived from early (E)- and late (L)-stage BD and healthy controls (HC). One-way ANOVA followed by Tukey post hoc, data expressed by mean ± SEM, *p < 0.05

**Fig. 4:**
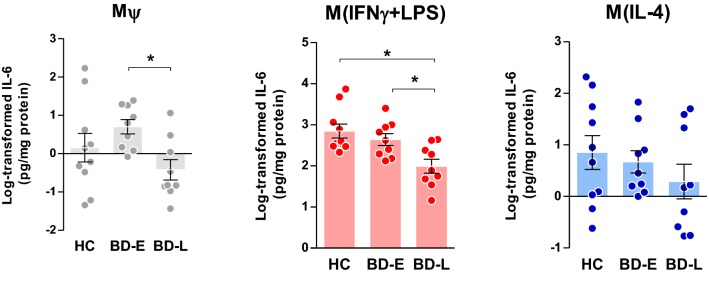
Levels of IL-6 secreted by different phenotypes of macrophages derived from individuals at early (E)- and late (L)-stage BD and healthy controls (HC). One-way ANOVA followed by Tukey post hoc, data expressed by mean ± SEM, *p < 0.05

Following post hoc analyses, M*ψ* of BD-L exhibited reduced secretion levels of IL-1β (p = 0.024, Fig. 2) when compared to controls, and TNFα (p = 0.027, Fig. 3) and IL-6 (p = 0.034, Fig. 4) when compared to early-stage patients. Overall, M(IFNγ + LPS) derived from BD-L secreted less pro-inflammatory cytokines compared to controls (IL-1β, p = 0.002; TNFα, p = 0.002; and IL-6, p = 0.003) and BD-E (IL-1β, p = 0.010; TNFα, p = 0.021; and IL-6, p = 0.028) (Figs. 2, 3, 4).

**Fig. 5:**
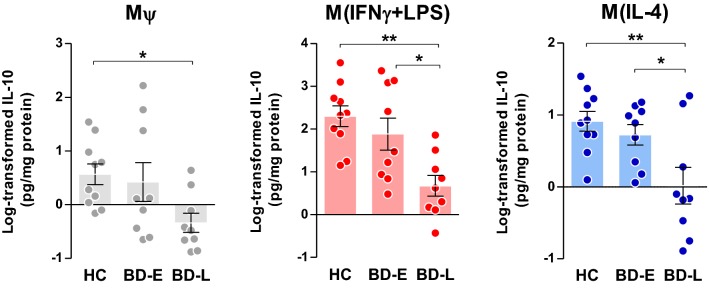
Anti-inflammatory IL-10 secretion by different phenotypes of macrophages derived from individuals with BD at early (E) and late (L) stages and healthy controls (HC). One-way ANOVA followed by Tukey post hoc, data expressed by mean ± SEM, *p < 0.05 and **p = 0.005

Different levels of the anti-inflammatory cytokine, IL-10, were secreted by the M*ψ* (F_2,25_ = 3.60, p = 0.042), M(IFNγ + LPS) (F_2,25_ = 8.56, p = 0.001) and M(IL-4) (F_2,25_ = 6.60, p = 0.005) phenotypes among groups. Tukey post hoc indicated that M*ψ* from late-stage patients produced less IL-10 than HC (p = 0.046), while M(IFNγ + LPS) and M(IL-4) phenotype secreted less than both BD-E (p = 0.020 and p = 0.033, respectively) and HC (p = 0.001 and p = 0.005, respectively) (Fig. 5).

## Discussion

The results of this preliminary and mechanistic study advance in the knowledge about the way that macrophages from BD patients across different stages of illness respond to stimuli, following classical (IFNγ and LPS) and alternative (IL-4) activation. To our knowledge, this is the first study showing a dysfunctional macrophage response against an inflammatory milieu in BD; and further investigating how these patterns change from early to more advanced stages of illness, given by the decreased secretion of inflammatory cytokines in late-stage patients. Albeit very preliminary, our data also suggest that a macrophage activity dysfunction occurs in parallel with the progression of the disease. In this way, our results showed a similar pattern of macrophage activation between patients in early stages of illness and healthy individuals, and, as the disease progresses, a significant decrease in macrophage activity was observed.

We used the secretion of IL-1β, TNFα, IL-6, and IL-10 as indexes of macrophage activity, as suggested by the literature (Becker et al. 2015). Our analysis provided a direct quantification of macrophages markers, given by cytokines secretion, across all induced phenotypes—M*ψ*, M(IFNγ + LPS) and M(IL-4)—and showed an association of changes in this secretion with different stages of illness. Interestingly, M(IFNγ + LPS) from patients at late stages of BD secreted lower amounts of IL-1β, TNFα and IL-6 as well as IL-10 compared to BD-E and HC. In M*ψ*, decreased levels of IL-1β and IL-10 were observed in BD-L derived cells compared to HC and TNFα and IL-6 when compared to BD-E. Furthermore, M(IL-4) from BD-L produced less IL-10 when compared to the other groups. Accordingly, our data sustain the hypothesis that during the early stages of the illness, macrophages that are exposed to inflammatory and anti-inflammatory stimuli present with intact function and polarize into what is known as classical (M1) and alternative (M2) phenotypes, respectively. However, in more advanced stages of BD, the secretion of macrophage markers is decreased, suggesting a decline in its activity. Corroborating these findings, a previous study showed that IL-10 was increased in the serum of early-stage BD patients, but not in the late-stage BD (Kauer-Sant’Anna et al. 2009). IL-10 can suppress the exacerbated immune response by neutralizing the deleterious effects of oxidative stress and proinflammatory cytokines, and increased levels may represent a compensatory response to counterbalance inflammatory process during BD, particularly at early stages (Modabbernia et al. 2013). On the other hand, the inability of macrophages to secrete adequate levels of IL-10, as observed in the BD-L, may represent a risk factor for poor prognosis. Furthermore, our findings support the concept of staging in BD showing a relationship between macrophage dysfunction and the chronic course of the illness.

Although data regarding the effects of BD on specific immune cell types are scarce, Knijff et al. (2007) reported that, after LPS stimulation, monocytes from BD patients secreted reduced levels of IL-1β and enhanced IL-6 production compared to the healthy subjects. Another independent study in major depression also showed that LPS-induced monocytes from patients with depression secreted lower levels of IL-1β, IL-6, and TNFα than those from the control group (Zhang et al. 2018). A recent report revealed that individuals with a history of early life adversity (e.g., young adults who had experienced parental loss in early life and were subsequently adopted) exhibited higher expression of the T cells markers and lower levels of IL-6 derived from ex vivo LPS-monocytes stimulation (Elwenspoek et al. 2017). Taken together these data suggest a reduction in macrophage functionality in stress-related disorders. Considering that macrophages are among the first innate line of defense against self and non-self-stimuli, it is plausible to speculate that mood disorders are associated with an immune tolerance phenomenon. If so, this would help to explain why individuals with psychiatric disorders are more likely to experience infections and immune-related disorders as well as have poor vaccine response (Barbuti et al. 2017; Zhang et al. 2018).

IL-6 and IL-1β are potent pleiotropic cytokines, with mainly proinflammatory effector function, that augment immune responses via induction of T cell activation, B cell proliferation, and differentiation, and stimulate acute phase protein release. In contrast to IL-6, increased IL-10 expression counteracts T cell responses, enhances injury-associated immunosuppression, and increases susceptibility to infection (Pfortmueller et al. 2017). Besides, these circulating cytokines can cross the blood–brain barrier and activate microglia through direct and indirect pathways which ultimately increases local production of inflammatory cytokines and oxidative stress mediators (Biesmans et al. 2013; Jakobsson et al. 2015). In this regard, Bayer et al. (1999) found increased microglial activation associated with HLA-DR expression—an MHC-II—in the prefrontal cortex (PFC) of affective disorder and schizophrenia patients at later stages, when compared to healthy individuals. Steiner et al. (2008) observed a marked microgliosis in the PFC and thalamus of individuals with depression and schizophrenia who committed suicide, suggesting a strong association between microglial activation and the severity of psychiatric disorders. Although brain microglia and macrophages and blood-derived monocytes have different embryonic origins (Goldmann et al. 2016), under a state of chronic sterile inflammation, as it occurs in BD, triggered by social defeat stress (Wohleb et al. 2011) or TNFα (D’Mello et al. 2009), immune-activated cells can migrate to the CNS. This cellular pathway mechanism has been elucidated in depression and sheds light into how inflammatory pathways from the periphery can transmit signals to the brain through the trafficking of preferentially bone-marrow derived monocytes, to brain vasculature and parenchyma. These observations suggest that common inflammatory markers from the innate immune system are present in the CNS and the periphery blood.

Furthermore, basal expression of innate receptors in monocytes from individuals with BD, such as Toll-like receptors (TLRs)-1, -2 and -6 are augmented under static conditions, but associated to reduced secretion of cytokines after stimulation (Wieck et al. 2016). Of interest, in previous studies conducted by our group, we observed increased DAMPs, which are endogenous signals released during cell stress, damage or death, in the serum of patients with BD (Stertz et al. 2015). DAMPs, in its turn, may bind to the TLRs, activating several signaling pathways and culminating in a sterile inflammation response (Stertz et al. 2015). Macrophages orchestrate cellular responses against a complex and diverse range of insults via pattern recognition receptors such as TLRs and Nod-like receptors (NLRs). In this sense, the activation of macrophages through DAMPs, TLRs and NLRs leads to the secretion of inflammatory mediators such as IL-1β. Corroborating with this, an increase in DAMPs release and early apoptosis in PBMCs of patients with BD has already been described (Fries et al. 2014). Since macrophages are responsible for the clearance of these molecules through phagocytosis, accumulation of DAMPs in BD may be related to macrophage dysfunction. Besides, the inability of macrophages from BD patients in the late stages of illness to respond to TLR stimulation through DAMPs could represent a risk factor for increased susceptibility to infections.

The present study has several limitations. First, the inclusion of participants was done by convenience. Therefore, we could not statistically control for potential determinants of immune response alterations, such as intrinsic cell defects, altered microenvironment, inflammation, biological stress, nutritional state or extrinsic factors such as additional environmental factors. Second, functioning was the criteria used to discriminate BD patients into stages since there is no valid method that covers clinical characteristics and functional status. Third, all patients were on polypharmacy, and the immune-modulating effects of medications in a patient with BD may have influenced our results. Also, despite being an exploratory design with small sample size, we consider that our results are important for the development and testing of new hypothesis in further studies.

## Conclusion

**Fig. 6:**
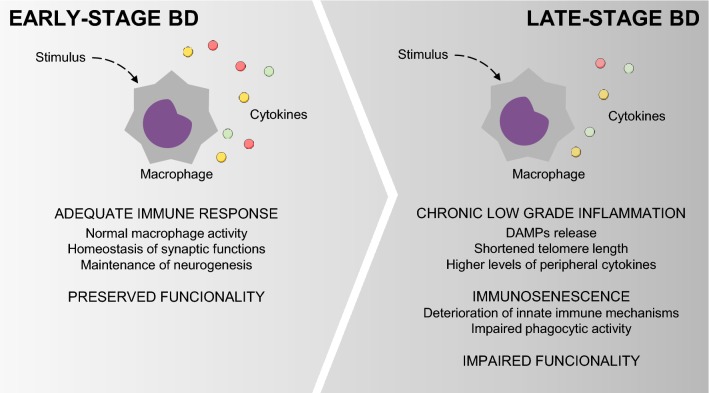
Macrophage activity impairment occurs in parallel with the progression of BD. Cell death, DAMPs release, telomere shortening, and proinflammatory mediators might lead to a chronic state of immune dysfunction, with a consequent decline in critical cellular processes. The immune system failures to counterbalance inflammatory responses, accelerates cell aging and leads to structural and neurocognitive changes with consequent loss of maintenance of tissue homeostasis and health

In conclusion, our findings support a causal attenuated response of macrophages for conversion, modulation, and function of classical (M1) and alternative (M2) phenotypes from monocyte-derived macrophages, which was more evident in the late stages of BD. Probably, systemic and environmental changes associated with an increase in cell death and DAMPs release may accelerate cell aging that, in turn, can promote a chronic state of immune dysfunction with consequent loss of maintenance of tissue homeostasis and health (Fig. 6). Further studies are required to investigate molecular mechanisms that may mediate macrophage dysfunction (e.g., PD-1), which may also contribute to a potential novel therapeutic target in BD.

## Data Availability

The datasets used and/or analyzed during the current study are available from the corresponding author on reasonable request.
